# Florbetaben PET in the Early Diagnosis of Alzheimer's Disease: A Discrete Event Simulation to Explore Its Potential Value and Key Data Gaps

**DOI:** 10.1155/2012/548157

**Published:** 2012-12-26

**Authors:** Shien Guo, Denis Getsios, Luis Hernandez, Kelly Cho, Elizabeth Lawler, Arman Altincatal, Stephan Lanes, Michael Blankenburg

**Affiliations:** ^1^United BioSource Corporation, Health Economics-Modeling and Simulation, 430 Bedford Street, Suite 300, Lexington, MA 02420, USA; ^2^Massachusetts Veterans Epidemiology Research and Information Center (MAVERIC) and VA Boston Healthcare System, 1400 VFW Parkway, West Roxbury, MA 02132, USA; ^3^Division of Aging, Department of Medicine, Brigham and Women's Hospital, Harvard Medical School, 1620 Tremont Street, Boston, MA 02120, USA; ^4^Boston University School of Public Health, 715 Albany Street, Boston, MA 02118, USA; ^5^United BioSource Corporation, Biostatistics, 430 Bedford Street, Suite 300, Lexington, MA 02420, USA; ^6^Bayer HealthCare Pharmaceuticals, Global Market Access - General Medicine, 178 Müllerstraße, 13353 Berlin, Germany

## Abstract

The growing understanding of the use of biomarkers in Alzheimer's disease (AD) may enable physicians to make more accurate and timely diagnoses. Florbetaben, a beta-amyloid tracer used with positron emission tomography (PET), is one of these diagnostic biomarkers. This analysis was undertaken to explore the potential value of florbetaben PET in the diagnosis of AD among patients with suspected dementia and to identify key data that are needed to further substantiate its value. A discrete event simulation was developed to conduct exploratory analyses from both US payer and societal perspectives. The model simulates the lifetime course of disease progression for individuals, evaluating the impact of their patient management from initial diagnostic work-up to final diagnosis. Model inputs were obtained from specific analyses of a large longitudinal dataset from the New England Veterans Healthcare System and supplemented with data from public data sources and assumptions. The analyses indicate that florbetaben PET has the potential to improve patient outcomes and reduce costs under certain scenarios. Key data on the use of florbetaben PET, such as its influence on time to confirmation of final diagnosis, treatment uptake, and treatment persistency, are unavailable and would be required to confirm its value.

## 1. Introduction

Alzheimer's disease (AD) is a fatal and progressive neurodegenerative disorder that affects millions of people worldwide [1]. It is currently the sixth leading cause of death in the United States (US), with about 80,000 deaths associated with AD in 2009 [2], and has imposed substantial burden on patients, their caregivers, medical care payers, and society as a whole [3]. With the baby-boomer generation aging, the economic and humanistic burdens caused by AD are expected to grow considerably if effective interventions cannot be discovered in time.

There is still no cure for AD. Cholinesterase inhibitors and memantine are the only major pharmacological treatments currently available to slow the symptoms associated with disease progression. Clinical studies have demonstrated modest benefits of these treatments in improving the symptoms related to AD [4, 5]. Prior studies have also indicated that identifying patients with AD and treating them at an early stage could result in cost savings and health benefits compared with treating them at a later stage due to the absence of early assessment [6, 7]. Current diagnostic tools in the early diagnosis of AD, however, are imprecise and detect the disease only based on the symptoms. Misdiagnosis and delayed diagnosis of AD, therefore, have been commonly reported [8–10].

The current diagnosis of AD is mainly based on clinical grounds according to the guidelines developed jointly by the National Institute of Neurological and Communicative Disorders and Stroke and the Alzheimer's Disease and Related Disorders Association (NINCDS-ADRDA). Although sensitive for AD, the NINCDS-ADRDA guidelines have poor specificity [11], which could lead to a great number of false positive cases and, consequently, unnecessary treatment. The growing knowledge in the use of biomarkers of AD pathology is very likely to provide physicians with new tools to not only improve the differential diagnosis of AD but also identify AD at an earlier stage and even before symptoms occur. The International Working Group for New Research Criteria for the Diagnosis of AD has recently revised the definition of AD to include both the predementia (or prodromal AD), referring to the early symptomatic phase of AD that is still not sufficiently severe to affect instrumental activities of daily living, and dementia (or AD dementia) phases. It has also recommended that the diagnosis of AD relies on a dual clinicobiological process that entails the evidence of both specific cognitive impairments and biomarkers of AD pathology that can include retention of amyloid tracers on positron emission tomography (PET); cerebral spinal fluid (CSF) beta-amyloid, total tau, and phospho-tau; medial temporal lobe atrophy (MTA) on magnetic resonance imaging (MRI); and/or temporal/parietal hypometabolism on fluorodeoxyglucose (FDG-) PET [12]. 

Florbetaben, a beta-amyloid tracer, is one of the novel diagnostic tools that can be used to detect neuropathological changes related to AD in vivo. It binds to beta-amyloid plaques and can be detected using a PET scan. The predictive values of florbetaben PET are currently under a phase III assessment, but preliminary phase II data have shown promising results in discriminating AD from other dementias and healthy controls [13–15]. The implications of this diagnostic tool in detection, diagnosis, and treatment of AD in actual clinical practice could be substantial once it is approved for use. However, due to increasing health resource constraints, the widespread use of florbetaben PET in the future clinical practice would depend not only on its clinical value but also its economic impact. Thus, the purposes of this study were to develop an early exploratory economic model assessing the potential clinical and economic value of florbetaben PET in the diagnosis of AD and to identify key value drivers as well as data gaps, which will direct the future research to support the future economic assessment of this technology. The design of this model and the findings from this assessment could also be very useful to guide further research assessing the cost effectiveness of other biomarkers, in the diagnosis of AD. 

## 2. Materials and Methods

The model explores the potential clinical and economic consequences of using florbetaben PET in the usual diagnostic process for the diagnosis of AD from both the US payer and societal perspectives. Usual diagnostic care refers to a period of diagnostic work-up during which specific diagnostic tests are performed over a series of medical visits to obtain the needed information for confirmation of a specific type of dementia diagnosis. Different combinations of diagnostic tests and assessments, along with other tests of pathophysiological (e.g., amyloid tracers and total tau) and/or topographical makers (e.g., FDG and MTA), received by patients during the diagnostic work-up period, could have different predictive values in terms of differentiating AD from other forms of dementia; there are numerous possible combinations of the tests and assessments that can be received by patients during the work-up period. Thus, for the purpose of simplicity and availability of the existing data to inform the predictive values of each specific combination of tests received by patients, the possible combinations of the diagnostic tests in this model were categorized into the following algorithms: clinical guidelines alone (e.g., NINCDS-ADRDA) and clinical guidelines plus one of the following tests: (1) MRI of MTA, (2) computerized tomography (CT) of MTA, (3) FDG-PET, (4) single photon emission computer tomography (SPECT), (5) CSF of beta-amyloid, total tau, phospho-tau, or beta-amyloid plus total tau, and (6) PET of florbetaben beta-amyloid tracers. Patients in the nonflorbetaben group (or called usual diagnostic care group hereafter) can be proportionally assigned to any of these diagnostic algorithms, except for the clinical guidelines plus florbetaben PET, to obtain an aggregate measure of sensitivity and specificity associated with the diagnostic algorithms assigned. 

This model was implemented as an individual patient simulation using discrete event simulation (DES) [24]. This modelling technique was selected mainly due to its capability to track events experienced by patients over the course of simulation and to use them, along with other relevant patient and disease characteristics, to predict the course of disease progression over time. This feature, along with other advantages, has led to increasing use of DES in assessing the cost effectiveness of treatments in patients with AD [7, 25, 26].

### 2.1. Model Concept

**Figure 1:**
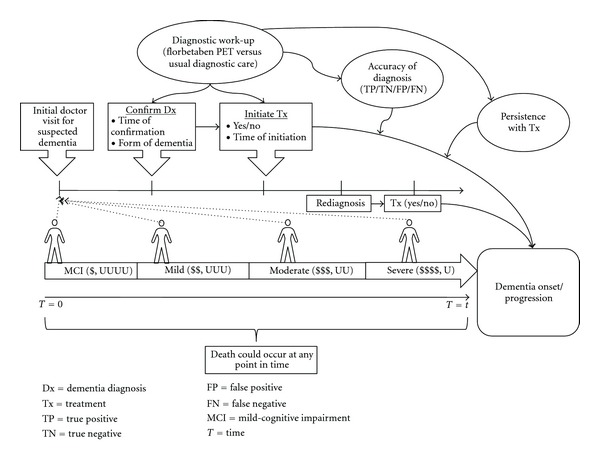
Schematic representation of the model concept.

Figure 1 shows the simplified schematic representation of the model concept. The target model population includes patients with some form of dementia who present to doctor offices for the first time, and those with cognitive problems due to other illnesses, such as depression or schizophrenia, were not considered in this analysis. Patients could make their initial doctor visit in either the predementia or dementia phase of the disease. The latter can be further divided into several levels of severity based on the Mini-Mental State Examination (MMSE) scores. Patients in the dementia phase are assigned an underlying pathological cause of dementia, which can be one of the following types of dementia: AD, mixed AD, vascular dementia (VaD), Lewy body dementia (LBD), frontotemporal dementia (FTD), and mixed non-AD. Those in the predementia phase are also assigned an underlying cause of either prodromal AD or nonprodromal AD, which could be any of the non-AD dementia listed above if the patient progresses to the dementia phase. Relevant patient and disease characteristics, such as age, gender, race, location of care, baseline severity, and comorbidities, are also assigned to each patient at the beginning of the simulation conditional on their underlying cause. 

Each patient in the model undergoes a period of diagnostic work-up following their initial doctor visit. At the end of the diagnostic work-up period, all the predementia patients are assumed to be correctly confirmed with a predementia diagnosis, and those assigned to the florbetaben group are given a PET scan, with dementia treatment, such as cholinesterase inhibitors and memantine, initiated for those with a positive AD result. For those assigned to the usual diagnostic care group, all or some proportion of the patients may receive dementia treatment without screening after their predementia diagnosis is confirmed. 

For the dementia patients, all patients are confirmed with a specific type of dementia at the end of diagnostic work-up, but the correctness of the diagnosis is dependent on the predictive values of the diagnostic algorithm assigned to the patient. Dementia treatment is initiated to all dementia patients in the florbetaben group with a positive AD result, but not to those with a negative result at the time of diagnosis confirmation. For those in the usual diagnostic care group, some dementia patients also have a chance to receive dementia treatment at the time of confirmation, depending on the result of diagnosis. Patients with a positive AD result but not treated at the time of confirmation could receive dementia treatment at a later time. 

Dementia treatment may delay progression to the dementia phase for patients with predementia. When a patient develops dementia, treatment may slow progression to a more severe stage of the disease, as well as need for institutional care, but the effect of treatment could be negatively impacted by misdiagnosis and nonpersistence with treatment. Disease progression in this model was modelled through the interrelated changes in 3 domains over time: cognition, using MMSE scale; behaviour, using the Neuropsychiatric Inventory (NPI) scale; function, using both the activities of daily living (ADL) and instrumental activities of daily living (IADL) scales. In this model, it is assumed that only those with prodromal AD, AD, or mixed AD as the underlying cause of dementia would benefit from the treatment with cholinesterase inhibitors or memantine. Treatment initiated in patients with non-AD would only have an impact on treatment costs.

Disease progression continues during the diagnostic work-up period. Shortening the time required to correctly confirm a diagnosis would allow appropriate treatments to be initiated at an earlier stage of the disease and thus could result in greater health benefits at lower costs. Use of florbetaben PET in this model could directly influence 4 major areas: (1) time required to confirm a diagnosis, (2) accuracy of diagnosis, (3) proportion of patients receiving appropriate dementia treatments at confirmation, and (4) persistence with treatment. Each of these impacts is associated with specific clinical and economic consequences. Finally, this model allows those who are misdiagnosed to be correctly rediagnosed at a later time and receive treatment. Death can occur at any point in time, and is dependent on patient age, gender, underlying cause of dementia, and stage of the disease (i.e., predementia and dementia phases). A simplified model flow diagram showing how patients are simulated is displayed and explained in Appendix A.

### 2.2. Data Sources

The primary data source used to populate this model was based on the administrative databases from New England Veterans Healthcare System (VISN 1) from January 1, 2002 through December 31, 2009 (fiscal years 2002–2009). Specific analyses from the VA VISN 1 data were performed to obtain the majority of the model inputs. Detailed information on the VA VISN 1 data can be seen in Appendix B. Model inputs which could not be obtained from the VA VISN 1 data were supplemented with data from literature, public databases, and assumptions. 

#### 2.2.1. Model Settings

A reference-case analysis was performed based on 1,000 simulated patients per group per run for a total of 10 replications. The model time horizon for the reference-case analysis was lifetime, which is commonly used for the assessment in this therapeutic area. Costs and benefits were discounted at 3% per annum [27]. Additionally, disease severity was divided into five levels based on the ranges of MMSE scores previously used by the United Kingdom National Institute for Health and Clinical Excellence [28]. 

#### 2.2.2. Patient and Disease Characteristics at Baseline

Data used to create the model population in the simulation were mainly obtained from the VA VISN 1 data, supplemented with data from literature. Among the patients with a confirmed diagnosis, 68% of them were confirmed with a dementia diagnosis and 32% with a predementia diagnosis. For those with a dementia diagnosis, 67% had a confirmed diagnosis of AD or mixed AD, 28% had a VaD, and the remaining 5% had other dementia diagnoses, such as LBD, FTD, and mixed non-AD. For those diagnoses with predementia, 65% were assumed to have prodromal AD, which was estimated based on a chart review of a subset of these patients. The mean age at initial diagnosis was about 78 years for patients with predementia and 82 years for patients with dementia. Data used to assign gender to the model populations (about 30% male) were obtained from literature [29–31] rather than from the VA VISN 1 data as almost all patients in the study cohort were male. 

Baseline MMSE scores were obtained from a chart review of a subset (*n* = 229) of the VA VISN 1 study cohort, indicating that more than 60% and 80% of the AD and non-AD patients, respectively, had the MMSE scores above 20 at initial diagnosis. As the NPI, ADL, and IADL scores were not available from the VA VISN 1 data, assumptions were made to assign the baseline scores for these measures consistent with the distribution of severity level based on the baseline MMSE scores. 

#### 2.2.3. Diagnostic Algorithms and Corresponding Predictive Values

The proportions of patients in the usual diagnostic care group undergoing a specific diagnostic algorithm were obtained from the VA VISN 1 data (Table 1). Table 1 also shows sensitivity and specificity by severity of the disease for each diagnostic algorithm. Data, except for florbetaben PET, were from a recent meta-analysis conducted by Bloudek and colleagues [11]. The sensitivity and specificity of florbetaben PET were supplied by the manufacturer of florbetaben tracers based on its internal analyses of preliminary phase II data as well as published data from other amyloid tracers [13–15].

#### 2.2.4. Time to Confirmation of Diagnosis

The amount of time taken to confirm a specific type of dementia diagnosis from the initial office visit under usual diagnostic care was predicted using parametric equations derived from the VA VISN 1 data.  Table 2 shows the equation for each diagnosis. Predictors with a positive coefficient indicate a longer time to confirmation of diagnosis as the values of the predictors increase and vice versa. The VA VISN 1 data show that the average time to diagnosis confirmation was about 5.1 months for AD or mixed AD, 6.1 months for VaD, 5.7 months for other non-AD, and 5.5 months for predementia diagnosis. In the reference-case analysis, use of a florbetaben PET was assumed to lead to a 50% reduction in time to diagnosis confirmation under usual diagnostic care. As the evidence to support this still does not exist, extensive sensitivity analyses were used to assess the impact of these assumptions on predicted outcomes. 

#### 2.2.5. Dementia Treatment

Dementia medications can be initiated at either the time of diagnosis confirmation or a later time for those patients in the usual diagnostic care group (Table 3). The majority of the patients were treated with donepezil based on the VA VISN 1 data. For patients in the usual diagnostic care group who were not treated at diagnosis confirmation, their time to treatment initiation was predicted using the parametric equations derived from the VA VISN 1 data (Table 2), indicating a median time of 28 months to treatment initiation for the dementia cohort and 42 months for the predementia cohort. 

Patients on any dementia treatment may discontinue over time.  Table 2 shows the parametric equations used to predict the time to discontinuation based on the VA VISN 1 data, indicating a median time of 36 months to treatment discontinuation for patients with dementia and 42 months for patients with predementia. The latter included the duration of treatment during the dementia phase. In the reference-case analysis, it was assumed that use of florbetaben PET could reduce the risk of discontinuation by 50%. This assumption, also tested in the sensitivity analyses, is based on the rationale that the improved accuracy of florbetaben PET may increase physicians' and patients' level of confidence in the diagnosis and therefore encourage them to persist longer with treatment. 

In this model, dementia treatment can also be forced to stop under some conditions specified by the users. In the reference-case analysis, patients with dementia were allowed to receive dementia treatment for lifetime as long as their MMSE scores were greater than 10; patients with predementia were assumed to receive treatment for no longer than five years if the patient did not covert to the dementia phase. 

#### 2.2.6. Disease Progression

Patients with predementia may convert to the dementia phase at a later time. Data on time to conversion, also from the VA VISN 1 data, were fitted to a Weibull function (Table 2). Based on the VA VISN 1 data, the median time to conversion was about 35 months. In the reference-case analysis, it was assumed that dementia treatment during the predementia phase would reduce the risk of conversion by 50% among those patients with prodromal AD, although the evidence to support such clinical benefits remains uncertain. This assumption was made to also assess the potential benefits of using florbetaben PET to screen predementia patients for treatment if a new effective treatment becomes available in the near future. Treatment for those patients with nonprodromal AD would have no clinical benefit but would have a cost impact. 

Disease progression for patients with AD or mixed AD was modelled based on the interrelated changes in MMSE, NPI, ADL, and IADL over time. Data used to simulate the disease progression were based on the predictive equations (Table 2) obtained from a study conducted by Getsios and colleagues [25]. On the other hand, disease progression for patients with non-AD dementia was simulated only based on changes in MMSE over time due to lack of data on NPI, ADL, and IADL. The same equation from Getsios and colleagues was used without any adjustment as few studies have shown that patients with other non-AD dementia, except for FTD, have similar rates of decline in cognition to those with AD [32–35]. For patients with FTD, the annual rate of change in MMSE scores was adjusted by −4.4 points [34].

#### 2.2.7. Time to Institutional Care

Two Weibull equations derived from the VA VISN 1 data were used to predict the time to institutional care for dementia and predementia patients (Table 2). Age was the only predictor in the equation for patients with predementia, indicating that the older patients are more likely to need institutional care than younger patients. On the other hand, predictors in the equation for patients with dementia include type of dementia diagnosis, time to confirmed diagnosis, and use of any dementia medication. Patients diagnosed with AD were more likely to need institutional care than patients diagnosed with other types of dementia; a longer time to confirmation of diagnosis was associated with a shorter time to institutional care; the and use of any dementia medication was associated with a longer time to institutional care. 

#### 2.2.8. Time to Death

Time to death was predicted using two gender-specific Gompertz functions derived from the US Life Table based on patients age 55 years and above (Table 2) [36]. These baseline risks were adjusted with hazard ratios of 1.48 for patients with predementia, 2.84 for patients with AD or mixed AD, and 2.69 for patients with non-AD dementia [37].

#### 2.2.9. Costs and Resource Uses

Cost inputs and their corresponding sources are shown in Table 4. Cost items considered in this model included costs of diagnostic work-up, imaging and biomarker tests, dementia medications, and medical and nonmedical care for predementia and dementia, including caregiver time. All cost inputs used 2011 values. Detailed information on cost inputs can be viewed in Appendix C. 

#### 2.2.10. Utilities

The model estimates utilities for both patients and their caregivers. Health utilities for patients with AD were estimated based on a published regression equation shown in Table 2 [38]. Health utilities for patients with non-AD were estimated by adjusting 0.006 lower compared to their AD counterparts [39]. A utility weight of 0.82 was used for patients with predementia [40]. Caregiver utilities were predicted with an equation (Table 2) from a report by Getsios and colleagues [25]. 

## 3. Results

### 3.1. Reference-Case Analyses

#### 3.1.1. At Baseline

Of the 1,000 simulated patients, 32% had predementia and 68% dementia, replicating the underlying input data. Of those patients with predementia, 65% had prodromal AD as the underlying cause. Patients with predementia had a mean age of 78 years, and mean scores of 27.5 on the MMSE, 2.5 on the NPI, and 10.1 on both ADL and IADL scales. On the other hand, for those with dementia, 67% had AD or mixed AD as the underlying cause, 28% had VaD, and 5% had LBD, FTD, or other mixed no-AD dementia with a mean age of 82 years and mean scores of 21.9 on the MMSE, 16.3 on the NPI, 29.7 on ADL, and 29.1 on IADL scales. 

#### 3.1.2. Predementia Cohort

The reference-case analysis shows that the average time to confirmation of predementia diagnosis was 4.64 months under usual diagnostic care, which was slightly lower than the time indicated by the VA VISN 1 data due to death and early conversion to dementia during the diagnostic work-up period, and 2.49 months with use of florbetaben PET. 

Due to death and early conversion, only about 92% (*n* = 295) of the predementia patients in the florbetaben group received the scan. Of these, 62% had a positive result, consisting of 58% true positive cases and 4% false positive cases (i.e., patients had non-AD dementia, but misdiagnosed as having AD), and thus received dementia treatment. For the remaining 38% of patients with a negative result, including 31% true negative and 7% false negative cases (i.e., patients had AD, but misdiagnosed as having non-AD dementia), dementia treatment was not initiated. The florbetaben group had a mean life expectancy of 0.10 years longer than the usual diagnostic group (Table 5), which was due to the delay in conversion to dementia in which the risk of death was greater. On average, patients in the florbetaben group had better other clinical outcomes than patients in the usual diagnostic groups in terms of time staying in predementia phase, time to institutional care, time spent in severer stages of the disease, and caregiver time (Table 5). These resulted in net discounted cost savings of $12,374 per patient over lifetime in direct medical care, $643 in caregiver time, and $13,018 in total cost. These savings were mainly due to reduction in several cost areas, with the greatest savings coming from reduced institutional care. Moreover, these clinical benefits also led to a net QALY gain (Table 5), making the use of florbetaben PET a dominant strategy in identifying and treating patients with prodromal AD under this reference-case scenario.

#### 3.1.3. Dementia Cohort

The reference-case results for the dementia cohort are also shown in Table 5. The average time to diagnosis confirmation was about 5.08 months with the usual diagnostic care group versus 2.66 months with the florbetaben group. Due to death, about 91% of the patients in the usual diagnostic care group and 95% in the florbetaben group completed the diagnostic work-up. More patients were misdiagnosed in the usual diagnostic care group, with 12% false-negative and 11% false-positive cases versus 7% false-negative and 3% false-positive cases in the florbetaben group. As expected, the average life expectancy for both groups was the same. On average, patients in the florbetaben group had better clinical outcomes than the usual care group (Table 5), leading to net discounted cost savings of $11,086 per patient over lifetime in direct medical care, $303 in caregiver time, and $11,389 in total cost. The majority of the cost savings resulted from reduction in costs associated with institutional care. QALYs for patients and caregivers were greater with the florbetaben group, but the differences were very small. This, nevertheless, still indicated that use of florbetaben PET in usual diagnostic care was a dominant strategy in the diagnosis of AD under this reference-case scenario. 

### 3.2. Deterministic Sensitivity Analyses

#### 3.2.1. Predementia Cohort

**Figure 2:**
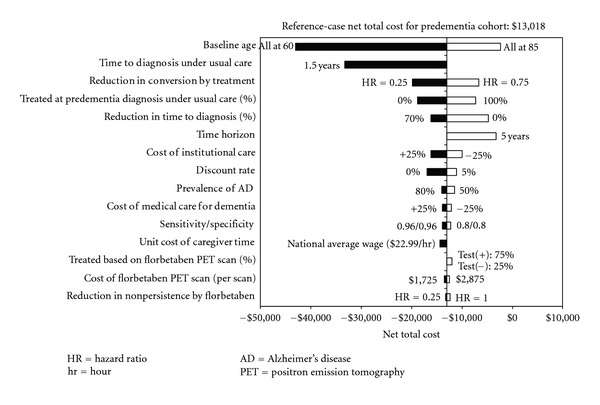
Results of univariate sensitivity analyses based on predementia cohort.

**Figure 3:**
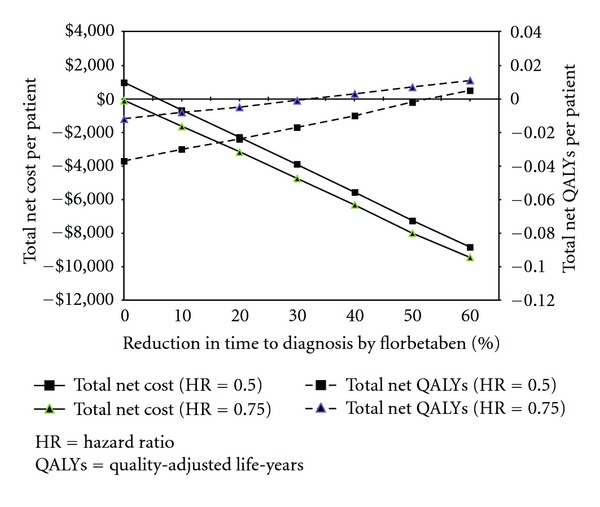
Impact of percent reduction in time to diagnosis on net cost and quality-adjusted life-years.

Figure 2 shows the top 15 model parameters with the greatest impact on total net costs. Given that the model results are very sensitive to variations in percent of patients treated in the usual diagnostic care group, treatment effect, and percent reduction in time to diagnosis confirmation by florbetaben PET, scenario analyses were conducted to understand the combined impact of the last two parameters on total net QALYs and costs in a scenario where all patients with a predementia diagnosis in the usual diagnostic care group were treated without screening.  Figure 3 shows the results of the scenario analyses, indicating that treating all predementia patients without screening could be a dominant strategy as compared to using florbetaben PET to screen patients with prodromal AD for treatment if there is no reduction in the time to confirmation of dementia diagnosis with florbetaben PET. The cost effectiveness of florbetaben PET would become more favourable if predementia treatment is less effective under this scenario. This is because more patients in the usual diagnostic care group are treated than in the florbetaben group. Assuming that a treatment could reduce the risk of conversion by 25% to 50%, it would require at least a 40% reduction in time to diagnosis confirmation in order for the use of florbetaben PET to be a dominant strategy under this particular scenario.

#### 3.2.2. Dementia Cohort

**Figure 4:**
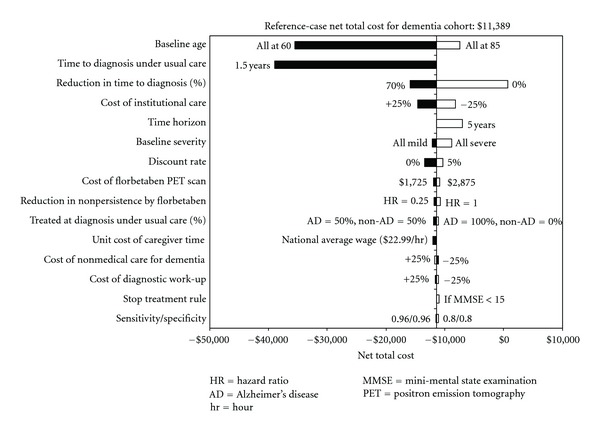
Results of univariate sensitivity analyses based on dementia cohort.

The top 15 parameters influencing the net cost based on the dementia patients were similar to those observed in the predementia cohort, but the levels of significance for some model parameters were somewhat different (Figure 4). The dominance of florbetaben PET could be altered if it had no impact on time to diagnosis confirmation. 

### 3.3. Probabilistic Sensitivity Analyses

**Figure 5:**
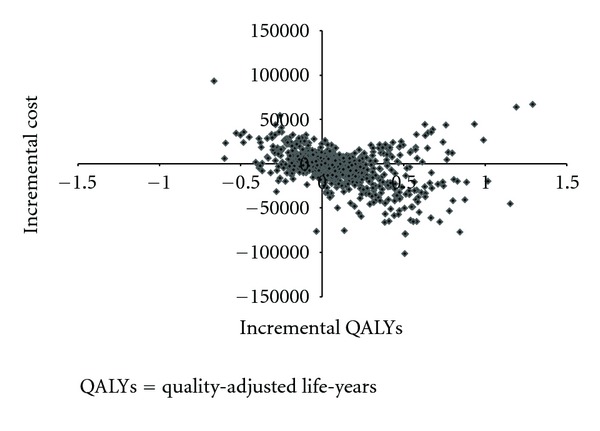
Incremental cost effectiveness plane for predementia cohort.

**Figure 6:**
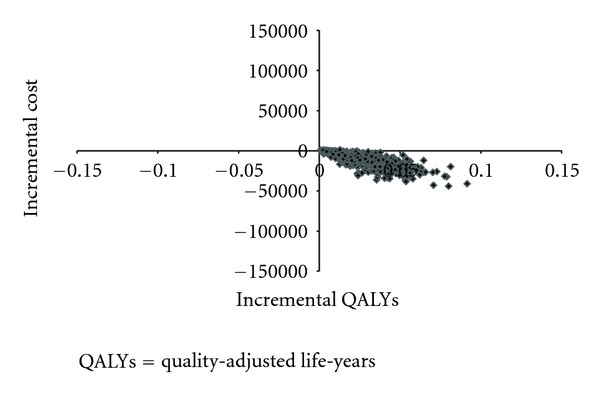
Incremental cost effectiveness plane for dementia cohort.

Detailed information on how the probabilistic sensitivity analyses were performed can be viewed in Appendix D. Figures 5 and 6 show the incremental cost effectiveness planes resulting from the probabilistic sensitivity analyses based on 1,000 replications. The cost effectiveness of florbetaben PET in the diagnosis of patients with prodromal AD is quite uncertain as the incremental cost effectiveness ratios (ICERs) scatter across 4 different quadrants (Figure 5). Based on the results of 1,000 replications, use of florbetaben PET among the predementia patients has net QALYs gained of 0.08, ranging from a worst case of −0.67 to a best case of 1.29, and an average net total cost of −$3,059, ranging from −$101,109 to $93,610. Using a willingness-to-pay threshold of $50,000 for one QALY, florbetaben PET would be considered cost effective in 58% of the replications. 

Unlike the results based on the predementia patients, almost all the ICERs based on the dementia patients spread in the fourth quadrant of the incremental cost effectiveness plane (Figure 6), indicating the dominance of florbetaben PET over usual diagnostic care. Based on the results of 1,000 replications, use of florbetaben PET among patients with dementia is associated with an average net QALY gain of 0.02, ranging from 0.0006 to 0.09, and a net total cost of −$9,525, ranging from −$44,210 to $1,117. With a willingness-to-pay threshold of $50,000 for one QALY, florbetaben PET is cost effective in 98% of the replications. 

## 4. Discussion

To our knowledge, this is the first model to assess the cost effectiveness of a biomarker in the early diagnosis of AD with DES to simulate the course of disease progression from predementia to dementia phase and its clinical management from initial diagnostic work-up to treatment initiation. The greater detail underlying the DES framework allows exploration of the potential value of biomarker use in the early diagnosis of AD, identification of major data gaps, and assessment of the uncertainty in outcomes associated with those gaps. As the model closely resembles the course of disease and its management at individual patient level, it inevitably requires richness of data to support the simulation. To deal with the data issue, we undertook a comprehensive analysis of longitudinal data from VA VISN 1 to characterize usual care pertaining to the diagnosis and treatment of AD and other forms of dementia in the US and to provide direct empirical estimates of various aspects of usual care of the diseases. With the use of advanced modelling technique, along with the support of comprehensive data from the VA VISN 1, this model provides a better understanding of how patients would be affected over time if a diagnostic biomarker like florbetaben PET tracer is used and which model parameters would have major influence on the model outcomes for specific patient groups. However, it should be noted that the results from the reference-case analysis are based on many important assumptions. Solid evidence to support or refute these assumptions is necessary before more conclusive estimates can be produced. 

The reference-case scenario indicates that use of florbetaben PET in the diagnosis of AD results in both health benefits and cost savings. The probabilistic sensitivity analyses suggest that such model outcomes are positive in the great majority of cases when florbetaben PET is used in patients with dementia but are subject to a greater uncertainty when used in patients identified with predementia. The greater uncertainty in the latter case is mainly due to lack of data on several critical model parameters. The deterministic sensitivity analyses indicate that improved accuracy of diagnosis alone would not be adequate to yield sufficient clinical benefits and cost offsets to justify the use of florbetaben PET in the diagnosis of AD. Other clinical benefits, especially if it would shorten the time taken to confirm a dementia or predementia diagnosis, are needed to further support its cost effectiveness.

The reasons the reduction in time to confirmed diagnosis is so important to the cost effectiveness of florbetaben PET are not only that early diagnosis could allow appropriate dementia treatment to be initiated at an earlier stage of the disease, but also that reduction in time to diagnosis confirmation has a direct beneficial impact on time to institutional care. The risk of needing institutional care would be reduced by about 12% for every 100-day reduction in time to diagnosis confirmation, as indicated by the analyses of the VA VISN 1 data. The causal relationship between them is still unclear. It is possible that early confirmation of diagnosis would allow patients and their family members to plan ahead and make needed adjustments to keep patients living independently as long as possible before they are sent to long-term-care facilities. Given that institutional care is costly, any minor delay to institutional care would have a meaningful impact on offsetting the cost of the scan. Data from a survey study, conducted alongside the florbetaben PET phase IIA trial [41] seem to also suggest that use of florbetaben PET can reduce the time to diagnosis confirmation. The survey shows that directly visualizing and evaluating a patient's amyloid burden in vivo highly increases the confidence of physicians in making their final diagnosis, suggesting that physicians are very likely to shorten the diagnostic work-up by eliminating additional examinations and “watchful” waiting period before sufficient symptoms are observed and consequently initiate appropriate treatment at an earlier stage. This could have a substantial positive impact on health and resource utilization benefits as there were approximately 72% and 23% of patients with predementia and AD diagnoses in the VA VISN 1 study cohort, respectively, who did not receive any dementia medication at confirmation until a median time of 42 and 28 months after confirmation. Use of florbetaben may shorten the delay to receipt of appropriate care substantially and result in better clinical and economic outcomes. 

Three additional important findings from the present analyses are worth mentioning as they may have important implications for future economic assessment of the florbetaben PET tracer in the early diagnosis of AD. First, a large discrepancy on time to confirmed diagnosis was found from the VA VISN 1 data when different approaches, that is, analysis of administrative data versus chart review of a subset of the study cohort, were used to quantify this duration. The estimated duration based on the analyses of the VA VISN 1 data may better capture the time taken to confirm a dementia diagnosis from the initial office visit because a diagnosis code would normally be recorded to represent the main complaint for a particular office visit. Yet, the estimated duration of 1.5 years based on the review of medical records should be a good proxy for the time to confirmation of diagnosis from the early signs and symptoms of AD as these could be recorded in the medical charts during the office visits for other medical problems. The implication of this discrepancy seems to pose a great opportunity for florbetaben PET tracers to identify patients with prodromal AD even at a much earlier stage if clinicians know when to use them. This would have a substantial, favourable impact on the cost effectiveness of florbetaben PET tracers.

Second, our deterministic sensitivity analyses show that younger patients would have a greater gain in net cost savings and QALYs from the use of florbetaben PET. This is due to a longer life expectancy in this population. In order to treat patients with prodromal AD or AD dementia at a younger age, screening general populations at younger ages seems to be a reasonable strategy. Although screening the general population for AD is not the focus of this assessment, the significant gain in economic and clinical benefits in the younger group from our analyses does suggest a promising possibility to support such an application for florbetaben PET tracers. This could also have valuable benefits to some of the patients who choose to know their disease propensity as early as possible despite the absence of effective treatments during the preclinical or predementia phase as it allows them to plan ahead with their life for personal and financial reasons [42, 43]. 

Third, as there is still no convincing evidence to support that treatment with cholinesterase inhibitors would yield any survival benefit, this model assumes that survival is independent of treatment effect, consistent with the assumption made in other published models [25, 26]. It is nevertheless important to highlight that if treatment does result in improved survival due to reduction in the rate of disease progression, use of florbetaben PET would yield more QALYs gained, but at the same time would result in higher overall costs, especially for long-term care. As future disease modifying treatments for AD may extend survival, this may have an important impact on the economic value of florbetaben PET and should be considered in future economic assessment of this technology. 

The present model has several major limitations. First, because the current analyses are mainly based on the data from the New England VA Healthcare System, the findings from this analysis may not be generalizable to patients in other regions of the VA Healthcare System, as well as in other healthcare systems outside of the VA system. Second, an external validation of this model to examine how well the model can predict the results observed in other studies has not yet been conducted due to lack of an appropriate external data source. However, the results of key model components, including disease progression during the dementia phase and time to clinical events shown in Table 2, were validated against the results from their respective data sources. A more complete external validation should be conducted when appropriate data source becomes available. Third, consistent with many other modelling studies in this therapeutic area [25, 26], continued treatment with dementia medication after 1 year was assumed to have a maintenance function only and no further treatment benefits in terms of delaying disease progression. Fourth, the model assumes that all patients who present to their doctor for memory complaints have some type of dementia. This may not be completely true as memory complaints or cognitive problems could be caused by other health problems, such as depression. Inclusion of these patients who in fact have no dementia may have some impact on the model results, depending on the prevalence of these conditions. Finally, the model, for the purpose of simplicity, assumed that all non-AD patients with a negative result at the end of diagnostic work-up would have their diagnosis correctly confirmed. This might have underestimated the benefits of florbetaben PET as it helps rule out AD as the underlying cause with a greater degree of certainty compared to other diagnostic tools, which may consequently help identify the true cause for non-AD dementia. 

## 5. Conclusions

This economic model provides a comprehensive framework to explore the potential clinical and economic value of florbetaben PET in the early diagnosis of AD among patients who present to their physicians' office for the first time due to cognitive complaints, to identify key value drivers as well as potential data gaps. Our exploratory analyses suggest that florbetaben PET has the potential to be a valuable tool in the diagnosis of AD as it would improve the health benefits of patients (with dementia as well as predementia) and their caregivers at a lower cost under certain scenarios. While the findings from the analyses to a large extent are supported by the data from a large longitudinal database and published literature, they rest also on many key assumptions and are subject to great uncertainty. Data on how the technology would impact clinical decision making and outcomes, such as time to confirmation of diagnosis, treatment uptake, and treatment persistency, will be needed to further substantiate its value.

## Figures and Tables

**Figure 7:**
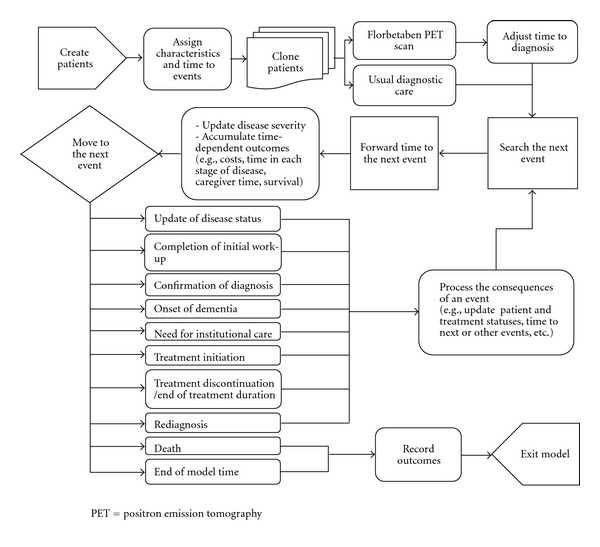
Model flow.

**Table 1:** Distribution of diagnostic algorithms and corresponding accuracy by severity (based on MMSE).

Diagnostic algorithm	%		Mild		Moderate and severe	
	Usual care	Florbetaben	Sensitivity	Specificity	Sensitivity	Specificity
Clinical guidelines only	73%	0%	87%	59%	77%	73%
Clinical guidelines with						
MRI of MTA	8%	0%	82%	66%	85%	80%
CT of MTA	19%	0%	80%	87%	80%	87%
FDG-PET	0%	0%	91%	75%	91%	86%
SPECT	0%	0%	79%	81%	68%	86%
CSF A*β*1-42	0%	0%	72%	75%	74%	79%
CSF A*β*1-42 + Ttau	0%	0%	86%	64%	84%	72%
CSF Ttau	0%	0%	77%	73%	82%	71%
CSF Ptau	0%	0%	77%	73%	82%	78%
Florbetaben PET	0%	100%	90%	90%	90%	90%

MRI: magnetic resonance imaging; MTA: medial temporal lobe atrophy; CT: computer tomography; PET: positron emission tomography; FDG: fluorodeoxyglucose; SPECT: single photon emission computer tomography; CSF: cerebral spinal fluid.

**Table 2:** Equations for prediction of time to events and disease progression.

Equation	Coefficient and predictor	SD/shape	Distribution
Time to confirmation of a diagnosis			
AD or mixed AD	4.571 + 0.327 Male + 0.252 MixedAD − 0.353 CKD	0.965	Lognormal
VaD	4.529 + 0.158 Diabetes + 0.203 Hypertension + 0.385 Stroke	1.005	Lognormal
Other non-AD dementia	6.558 − 0.029 Age + 1.554 Stroke + 0.654 LBD + 0.400 FTD	0.896	Lognormal
Predementia	3.981 + 0.009 Age − 0.243 CKD − 0.179 CVD	0.994	Lognormal
Time to treatment initiation if not started at diagnosis			
Dementia	7.149 − 0.022 Age + 1.056 VaD + 2.091 Other non-AD + 0.004 (time, in days, to confirmed diagnosis)	1.517	Lognormal
Predementia	18.781 − 0.150 Age	3.996	Lognormal
Time to treatment discontinuation			
Dementia	7.487 − 0.0008 (time to confirmed diagnosis)	0.922	Weibull
Predementia	7.122 + 0.443 (conversion to dementia)	1.131	Weibull
Time to conversion to dementia	Scale = 0.0212	0.952	Weibull
Rate of change in MMSE^a^	5.4663 − 0.4200 PM1 − 0.0042 PM2 + 0.1415 PM3 − 0.079 PrevRate + 0.07474 Age + δ_*i*_	N/A	N/A
Rate of change in NPI^b^	(5.74 − 0.64 Treatment + 0.03 Weeks − 0.59 NPI_base_ − 0.59 NPI Weeks + 0.24 NPI_recent_ − 1.74 White − 3.82 Black + 2.34 PsyMed + 0.12 MMSE_base_ − 0.22 MMSE_recent_ + δ_*i*_) ∗ 1.44	N/A	N/A
Rate of change in ADL	1.35 − 0.81 Treatment + 0.06 Weeks − 0.79 ADL_base_ + 0.71 IADL_previous_ + 0.12 MMSE_base_ + 0.09 Age + 0.81 PsyMed − 3.05 Black − 0.49 MMSE_recent_ + δ_*i*_	N/A	N/A
Rate of change in IADL	1.27 + 0.63 Treatment + 0.17 Weeks − 0.06 Treatment ∗ Weeks − 0.84 IADL_base_ − 0.002 IADL_base_ ∗ Weeks + 0.84 IADL_previous_ − 0.67 Male + 0.20 MMSE_base_ − 0.28 MMSE_recent_ − 0.16 ADL_base_ + 0.18 ADL_recent_ + δ_*i*_	N/A	N/A
Time to institutional care			
Dementia	9.883 − 0.02 Age + 0.295 VaD + 1.154 Other non-AD − 0.001 Time to confirmed diagnosis + 1.079 Dementia treatment	0.933	Weibull
Predementia	11.469 − 0.028 Age	1.373	Weibull
Time to death			
Male	Scale = −9.697	0.087	Gompertz
Female	Scale = −10.787	0.097	Gompertz
Patient utility	0.408 + 0.010 MMSE − 0.004 NPI − 0.159 Institutionalized + 0.051 Living with Caregiver	N/A	N/A
Caregiver utility	0.90 − 0.003 Age_CG_ + 0.03 Male_CG_ + 0.001 Male − 0.001 NPI − 0.001 ADL − 0.0004 IADL − 0.01 PsyMed	N/A	N/A

SD: standard deviation; AD: Alzheimer's disease; VaD: vascular dementia; LBD: Lewy body dementia; LTD: frontotemporal dementia; CKD: chronic kidney disease; CVD: cerebrovascular disease; MMSE: mini-mental state examination; NPI: neuropsychiatric inventory; ADL: activities of daily living; IADL: instrumental activities of daily living, CG: caregiver.

^a^PM represents patients' previous MMSE measurement, partitioned over the scale of MMSE. PrevRate is the patients' last known rate of decline. Age represents patients' age at baseline. δ_*i*_ represents a random intercept parameter.

^b^Treatment is dementia medication, Weeks represents weeks of followup in the simulation, NPI_base_ is the patient's baseline NPI, and NPI_recent_ is the patient's last NPI. White and Black are dummy variables for race, PsyMed is a dummy variable for patients on psychiatric medications at baseline, MMSE_base_ represents the patient's MMSE at baseline, and MMSE_recent_ represents the patient's current MMSE.

**Table 3:** Model parameters for treatments.

Parameter	Dementia		Predementia		Data source
	Usual care	Florbetaben	Usual care	Florbetaben
% of patients receiving dementia medication at diagnosis	N/A	N/A	28%	N/A	VA VISN 1 and user specification
If Dx = AD+	77%	100%	N/A	100%	
If Dx = non-AD	67%	0%	N/A	0%	
Distribution of dementia medication	**Dx = AD+**	**Dx = Non-AD**			VA VISN 1
Donepezil	63%	66%	76%		
Galantamine	25%	9%	6%		
Rivastigmine	5%	4%	1%		
Memantine	7%	21%	17%		
Maximum dementia treatment duration allowed, years	Life time		5		User specification
Stopping dementia medication if MMSE score is below 10	Yes		N/A		User specification

AD: Alzheimer's disease; Dx: dementia diagnosis; MMSE: mini-mental state examination; N/A: not applicable.

**Table 4:** Cost inputs.

Cost item	Value	Unit	Data source
Diagnostic work-up			
AD+	$5,120	Per year	
VaD	$5,885	Per year	VA VISN 1 and [16–18]
Other non-AD	$6,638	Per year	
Predementia	$6,187	Per year	
Imaging and biomarker tests			
MRI + MTA	$437	Per test	
CT + MTA	$300	Per test	
FDG-PET	$1,042	Per test	[16], manufacturer
SPECT	$596	Per test	
CSF	$304	Per test	
Florbetaben PET	$2,300	Per test	
Dementia medication			
Donepezil	$7.79	Per day	
Galantamine	$6.36	Per day	[19]
Rivastigmine	$6.11	Per day	
Memantine	$7.89	Per day	
Medical care for predementia	$5,548	Per year	[20]
Medical care for AD+			
Mild	$8,315	Per year	
Mildly moderate	$12,806	Per year	
Moderate	$12,806	Per year	[20]
Moderately severe	$18,526	Per year	
Severe	$23,227	Per year	
Nonmedical care for AD+			
Mild	$154	Per year	
Mildly moderate	$3,692	Per year	
Moderate	$12,166	Per year	[20]
Moderately severe	$14,209	Per year	
Severe	$23,355	Per year	
% of additional cost of care for non-AD relative to AD			
VaD	84%		[21]
Other non-AD	37%		
Institutional care	$373	Per day	[22]
Caregiver time	$7.25	Per hour	[23]
Caregiver burden for predementia	2.10	Hours per day	[20]
Caregiver burden for dementia			
Mild	2.10	Hours per day	
Mildly moderate	3.58	Hours per day	
Moderate	3.58	Hours per day	[20]
Moderately severe	3.76	Hours per day	
Severe	5.10	Hours per day	

AD: Alzheimer's disease; VaD: vascular dementia; MRI: magnetic resonance imaging; MTA: medial temporal lobe atrophy; CT: computer tomography; PET: positron emission tomography; FDG: fluorodeoxyglucose; SPECT: single photon emission computer tomography; CSF: cerebral spinal fluid.

**Table 5:** Reference-case results.

Outcome (per patient)	Predementia cohort (*n* = 320)			Dementia cohort (*n* = 680)		
	Usual care	Florbetaben	Net	Usual care	Florbetaben	Net
Survival, years	6.84	6.94	0.10	4.57	4.57	0.00
Time to confirmed diagnosis, months	4.64	2.49	−2.15	5.08	2.66	−2.42
Time in predementia, years	3.22	3.56	0.34	N/A	N/A	N/A
Time to institutional care, years	5.48	5.72	0.24	3.17	3.29	0.12
Time in severity, years						
Mild	3.53	3.82	0.29	0.56	0.60	0.04
Mildly moderate	0.46	0.44	−0.02	0.77	0.78	0.01
Moderate	0.48	0.45	−0.03	0.71	0.71	0.00
Moderately severe	0.42	0.40	−0.01	0.57	0.56	−0.01
Severe	1.96	1.83	−0.13	1.96	1.92	−0.05
Caregiver time, years	0.92	0.91	−0.01	0.77	0.76	−0.01
Costs (discounted)						
Total direct medical care	$301,599	$289,225	−$12,374	$314,156	$303,070	−$11,086
Caregiver time	$47,914	$47,271	−$643	$42,311	$42,008	−$303
Total	$349,514	$336,496	−$13,018	$356,466	$345,077	−$11,389
QALYs (discounted)						
Patients	3.53	3.68	0.15	1.75	1.78	0.03
Caregivers	4.29	4.41	0.12	2.59	2.60	0.01
Total	7.82	8.09	0.27	4.34	4.37	0.03
ICERs (discounted)						
Patients			Dominant			Dominant
Caregivers			Dominant			Dominant
Total			Dominant			Dominant

QALYs: quality-adjusted life years; ICERs: incremental cost-effectiveness ratios.

Note: inconsistency may occur due to rounding.

## Appendices

### A. Model Flow

A simplified flow diagram showing how patients are simulated in the model is displayed above. At the beginning of the simulation, the model creates 1,000 patients with different types of dementia, based on the prevalence of dementia for each type specified by the user, and assigns patient and disease characteristics conditional on their underlying cause. These characteristics include age, gender, race, baseline scores for Mini-Mental State Examination (MMSE), Neuropsychiatric Inventory (NPI), activities of daily living (ADL), instrumental activities of daily living (IADL), location of care (either home or institutional care), use of antipsychotics, comorbidities (i.e., chronic kidney disease, cardiovascular disease, diabetes, and hypertension), and caregiver's gender and age. These characteristics are used to predict the rate of disease progression and other model outcomes, such as time to death, time to institutional care, time to confirmation of diagnosis, costs of care, and health utilities. After the assignments of baseline patient characteristics and event times, each patient is cloned; one clone is assigned to the usual diagnostic care group and the other to the florbetaben group. The cloning step used in the simulation resembles a perfect randomization where both groups are comprised of exactly the same patients. For those assigned to the florbetaben group, their time to confirmation of diagnosis is updated based on a percent reduction in time to diagnosis specified by the user. Then, all patients are sent to the “search next event” module, where the next event for each patient is identified based on the event with the shortest time to occur. There are a total of 10 events conceptualized in this model, as shown in diagram above. After identifying the next event for a patient, the model then fast-forwards its clock to the next event time. Before the event is processed for its related consequences, such as updates of patient and treatment statuses and time to next event, all time-dependent outcomes—including survival, quality-adjusted life-years (QALYs), costs of care, caregiver time, time alive at each stage of the disease, and time spent in institutional care—are tallied and accumulated. After processing the consequences of the event, the patient proceeds to the “search next event” module again, and the same process is repeated until the patient dies or the model time ends see Figure 7.

### B. Database Analysis

The primary data source used to populate this model was based on the administrative databases (clinical, laboratory, and pharmacy databases) from New England Veterans Healthcare System (VISN 1) from January 1, 2002 through December 31, 2009 (fiscal years 2002–2009). The New England region consists of eight Veterans Administration (VA) Medical Centers and affiliated clinics providing inpatient and outpatient medical care. The VISN 1 pharmacy files were obtained from Information Resource Management, Boston, MA. ICD-9-CM diagnoses and laboratory data were captured by accessing the VA National Patient Care and Decision Support Systems administrative databases (Patient Treatment File and Outpatient Care File) located at the Austin Automation Center, Austin, TX. All database analyses were conducted at the Massachusetts Veterans Epidemiology Research and Information Center (MAVERIC), VA Boston Healthcare System, Boston, MA. The study was approved by the Institutional Review Board of the VA Boston Healthcare System.

Specific analyses from VISN 1 were performed to obtain the majority of the model inputs, including the following: (1) prevalence of dementia for each type and baseline patients and disease characteristics by dementia diagnosis, (2) time to confirmation of diagnosis from the initial visit, (3) resource use during the diagnostic work-up period, (4) proportion of the patients treated with cholinesterase inhibitors or memantine at time of diagnosis confirmation by dementia diagnosis, (5) time to treatment initiation if not treated at time of confirmation, (6) time to treatment discontinuation if treated, (7) time to conversion to the dementia phase for patients with predementia, and (8) time to institutional care. The study cohort for these analyses was based on 2,783 patients who were confirmed with a dementia (*n* = 1, 882) or predementia (*n* = 901) diagnosis within two years since the initial visit. The maximum two-year time to diagnosis confirmation was used to exclude those cases that may not actually have any dementia. The study cohort was selected from more than 19,000 subjects in the New England VA Healthcare System between January 1, 2002 and December 31, 2009 and who had one of the following dementia diagnoses Alzheimer disease (AD), senile and presenile of dementia of the AD type, vascular dementia (VaD), frontotemporal dementia (FTD), Lewy body dementia (LBD), mild cognitive impairment (MCI), memory loss, and cognitive deficits of cerebrovascular disease (CD-CVD). Additional inclusion criteria included patients at least 55 years of age at time of first dementia diagnosis, a 12-month baseline period in the VA system free of any dementia diagnosis prior to the first dementia diagnosis, and no record of cholinesterase inhibitors or memantine during the baseline period. Specific types of dementia diagnosis were confirmed if patients met one of the following two criteria: (1) at least two identical dementia ICD-9 diagnoses given by a recognized specialist, separated by a period of at least 30 days, and (2) at least one ICD-9 diagnosis of dementia accompanied by a recognized dementia medication, including donepezil, rivastigmine, galantamine, or memantine. Descriptive statistical analyses and derivations of parametric equations to predict time to events based on patient characteristics were performed.

Among the patients with a confirmed diagnosis (*n* = 2,783), 68% of them were confirmed with a dementia diagnosis and 32% with a predementia diagnosis. For those with a dementia diagnosis, 67% had a confirmed diagnosis of AD or mixed AD, 28% had a VaD, and the remaining 5% had other dementia diagnoses, such as LBD, FTD, and mixed non-AD. For those diagnoses with predementia, 65% were assumed to have prodromal AD, which was estimated based on a chart review of a subset of these patients. The mean age at initial diagnosis was about 78 years for patients with predementia and 82 years for patients with dementia. Comorbidities were highly prevalent among the study cohort due to their advanced age, with about 20% with a CVD, 27% with diabetes, 47% with coronary heart disease, 80% with hypertension, and 5% on antipsychotics at baseline.

### C. Cost Inputs

#### C.1. Diagnostic Work-Up

The costs of diagnostic work-up, including a series of office visits to primary care physicians and specialists, lab tests, and outpatient clinic visits, were estimated based on the time taken to confirm a diagnosis from the initial office visit. Data for healthcare resource use during the diagnostic work-up period were obtained for each dementia diagnosis from the VA VISN 1 data and were translated into costs by applying the unit costs from the Centers for Medicare & Medicaid Services (CMS) [16–18]. The estimated costs of diagnostic work-up for each diagnosis per year are shown in Table 4 of the main text.

#### C.2. Imaging and Biomarker Tests

The costs of brain imaging and biomarker analyses, a one-time cost based on the distribution of diagnostic algorithms assigned, were estimated using the cost data from CMS hospital outpatient fee schedule [16] (Table 4). The cost of florbetaben PET was set at $2,300 per test in the base-case analysis, which was suggested by the manufacturer of florbetaben.

#### C.3. Dementia Medication

Unit costs for dementia medications were based on the average wholesale price reported in the Red Book [19] (Table 4). The daily costs for these drugs were estimated based on their recommended dose and usage from the licensed labels.

#### C.4. Medical and Nonmedical Care for Predementia and Dementia

Costs of care were separated into costs of medical and nonmedical care. For patients with AD, these costs were obtained from a longitudinal study which followed 172 patients with probable AD for 4 years to examine the effects of patient dependence, measured by the Dependence Scale, on the following: (1) medical care costs including hospitalizations, outpatient treatments and procedures, and assistive devices; (2) nonmedical care costs, including overnight respite care, adult day care, and home healthcare; (3) informal caregiving time, including time used for ADL and for supervision [20]. As these outcomes of interest were reported in relation to the Dependence Scale (range = 0–15; higher scores indicate greater dependence), they were mapped onto the MMSE scores using the data from the same study to estimate the costs of medical and nonmedical care, as well as caregiver time, by level of severity, defined by MMSE scores. Table 4 shows the costs of medical and nonmedical care and the amount of caregiver time by each level of severity for patients with AD. As these costs were measured in 2005 values, they were inflated to 2011 values using the medical services component of the Consumer Price Index. The costs of care for patients with non-AD dementia were also estimated based on patients' levels of severity. The annual costs of care for patients with VaD and other non-AD were estimated by increasing 1.83 and 1.37 times the costs for patients with AD, respectively. These ratios were obtained from a study comparing the healthcare costs of community-dwelling patients with VaD (*n* = 678) and other non-AD dementia (*n* = 957) to patients with AD (*n* = 1,722) using Medicare HMO data during 1999–2002 [21]. The amount of caregiver time by level of severity for patients with non-AD was assumed to be the same as that for patients with AD due to lack of available data.

The cost of managing predementia in the US setting was not identified from the literature search. Although some studies were found for other countries [38, 44], they were not used in the analyses, as healthcare utilization patterns and costs vary significantly across countries. Thus, the average cost based on the Dependence Scale of 0-1 obtained from the study by Zhu and colleagues [20] was used to estimate the cost of care and caregiver time for patients with predementia (Table 4) [20].

### D. Analyses

Model outcomes for the base-case analysis included percent of patients misdiagnosed, time to diagnosis confirmation, number of predementia patients progressing to dementia, life-years, time alive at each severity stage, percent of patients needing institutional care, time to institutional care, caregiver time, costs, and QALYs for patients and caregivers. Deterministic sensitivity analyses, including one-way, subgroup, and scenario analyses, were performed to assess how the model outcomes vary in relation to changes in model parameters. Finally, in order to account for uncertainties from multiple key parameters, probabilistic sensitivity analyses were performed by simultaneously varying multiple parameters under the following assumptions. First, for the inputs which have no or little prior data to support, uniform distributions were used as it is a more conservative assumption. These parameters included the following: (1) sensitivity (80% for the lower bound–96% for the upper bound) and specificity (80–96%) of florbetaben PET, (2) percent reduction (0–100%) in time to diagnosis confirmation by florbetaben PET, (3) percent reduction (0–100%) in risk of conversion to dementia phase by treatment, and (4) percent reduction (0–100%) in treatment discontinuation by florbetaben PET. Second, for model inputs with prior data to support, beta distributions were used for categorical variables and normal distributions within 2 standard errors of the mean as the upper and lower bounds were used for continuous variables. For some of the parameters included in the probabilistic sensitivity analyses, standard errors were available from the parameter source data and thus used to measure parameter uncertainties. Where a standard error was not available for a selected parameter, we used 25% of the mean as an assumed standard error. Parameters included in the probabilistic sensitivity analyses and assumed to be beta distributed were as follows: (1) percents of the predementia and dementia patients treated at time of confirmation under usual diagnostic care, (2) ratios of cost of care for AD versus VaD and non-AD, and (3) patient and caregiver utilities for predementia patients. Parameters assumed to be normally distributed included the following: (1) all predictor coefficients for the parametric equations, including time to diagnosis, time to treatment initiation, time to treatment discontinuation, and time to institutional care; (2) coefficients for the treatment effects on rates of change in MMSE, NPI, ADL, and IADL over time; (3) hazard ratios of death (with log transformation) compared to general population for predementia, AD, and no-AD; (4) predictor coefficients for patient and caregiver utility equations for dementia patients; (5) cost of dementia medications; (6) costs of medical and nonmedical care for predementia and dementia; (7) cost of institutional care; (8) caregiver burden by severity level.
